# Tunicate cytostatic factor TC14-3 induces a polycomb group gene and histone modification through Ca^2+ ^binding and protein dimerization

**DOI:** 10.1186/1471-2121-13-3

**Published:** 2012-02-02

**Authors:** Kaz Kawamura, Kohki Takakura, Daigo Mori, Kohki Ikeda, Akio Nakamura, Tomohiko Suzuki

**Affiliations:** 1 Laboratory of Cellular and Molecular Biotechnology, Faculty of Science, Kochi University, Kochi 780-8520, Japan; 2 Department of Molecular and Cellular Pharmacology, Gunma University, School of Medicine, Maebashi, Gunma 371-8511, Japan; 3 Laboratory of Biochemistry, Faculty of Science, Kochi University, Kochi 780-8520, Japan

## Abstract

**Background:**

As many invertebrate species have multipotent cells that undergo cell growth and differentiation during regeneration and budding, many unique and interesting homeostatic factors are expected to exist in those animals. However, our understanding of such factors and global mechanisms remains very poor. Single zooids of the tunicate, *Polyandrocarpa misakiensis*, can give off as many as 40 buds during the life span. Bud development proceeds by means of transdifferentiation of very limited number of cells and tissues. TC14-3 is one of several different but closely related polypeptides isolated from *P. misakiensis*. It acts as a cytostatic factor that regulates proliferation, adhesion, and differentiation of multipotent cells, although the molecular mechanism remains uncertain. The Polycomb group (PcG) genes are involved in epigenetic control of genomic activity in mammals. In invertebrates except *Drosophila*, PcG and histone methylation have not been studied so extensively, and genome-wide gene regulation is poorly understood.

**Results:**

When Phe^65 ^of TC14-3 was mutated to an acidic amino acid, the resultant mutant protein failed to dimerize. The replacement of Thr^69 ^with Arg^69 ^made dimers unstable. When Glu^106 ^was changed to Gly^106^, the resultant mutant protein completely lost Ca^2+ ^binding. All these mutant proteins lacked cytostatic activity, indicating the requirement of protein dimerization and calcium for the activity. *Polyandrocarpa Eed*, a component of PcG, is highly expressed during budding, like TC14-3. When wild-type and mutant TC14-3s were applied in vivo and in vitro to *Polyandrocarpa *cells, only wild-type TC14-3 could induce *Eed *without affecting histone methyltransferase gene expression. Eed-expressing cells underwent trimethylation of histone H3 lysine27. *PmEed *knockdown by RNA interference rescued cultured cells from the growth-inhibitory effects of TC14-3.

**Conclusion:**

These results show that in *P. misakiensis*, the cytostatic activity of TC14-3 is mediated by *PmEed *and resultant histone modification, and that the gene expression requires both the protein dimerization and Ca^2+^-binding of TC14-3. This system consisting of a humoral factor, PcG, and histone methylation would contribute to the homeostatic regulation of cell growth and terminal differentiation of invertebrate multipotent cells.

## Background

Cell and tissue homeostasis are among the most important features of living organisms. In vertebrates, various types of extracellular molecules act as cell growth regulators. For example, angiostatin and endostatin are potent inhibitors of endothelial cell proliferation and angiogenesis [1,2]. They contribute to our understanding of in vivo cell growth homeostasis and therapeutic control of tumor angiogenesis [3]. Among invertebrates, many species have multipotent cells that undergo cell growth and differentiation during regeneration and budding [4,5]. Therefore, many unique and interesting homeostatic factors are expected to exist in invertebrates. However, our understanding of such factors and global mechanisms remains very poor.

**Figure 1:**
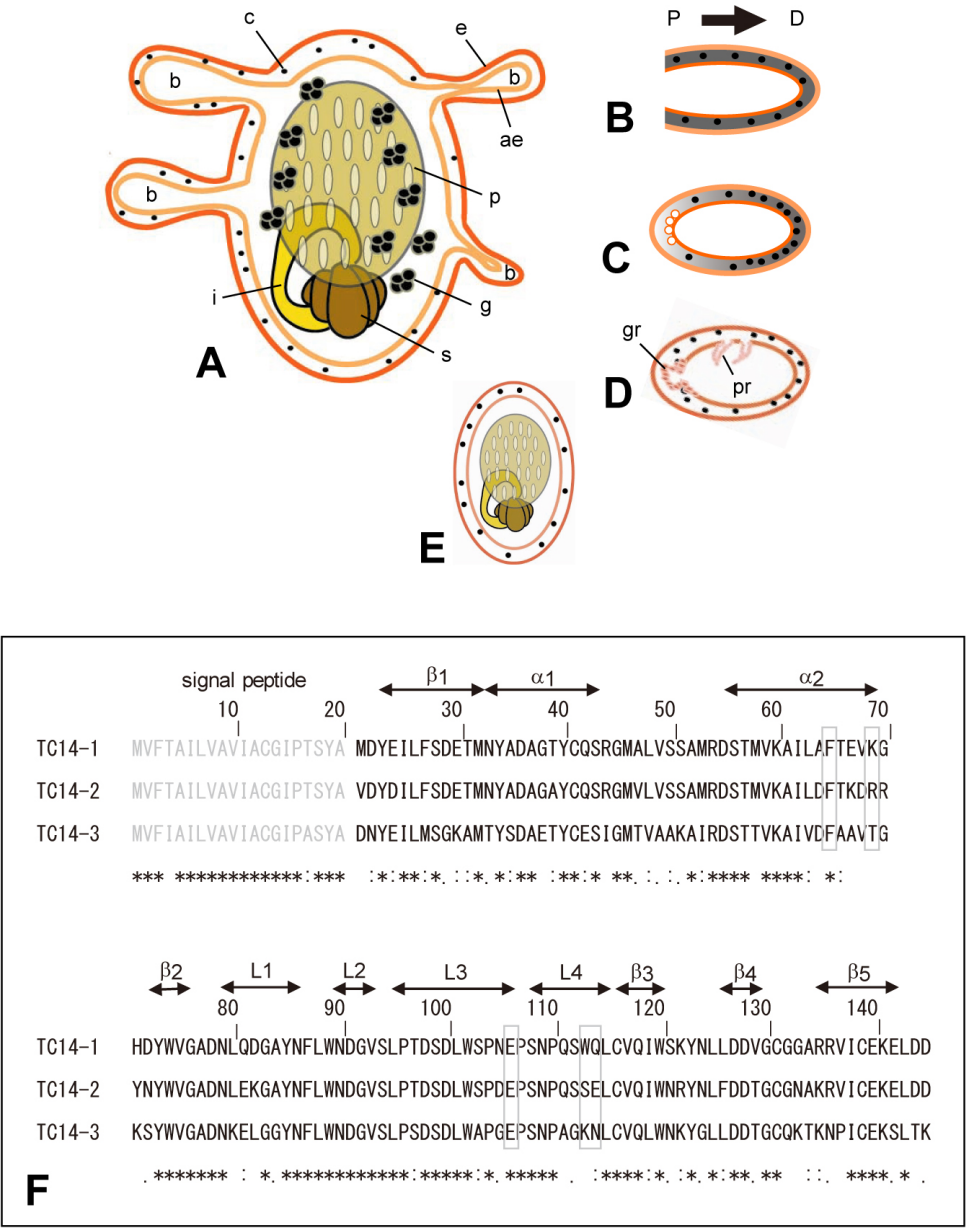
**Outline of the budding life cycle in *Polyandrocarpa misakiensis *(upper) and multiple alignment of TC14s (lower)**. (A)Adult zooid and buds (b) protruding from the parent body wall that consists of the epidermis (e), atrial epithelium (ae), and intervening coelomic cells (c). g, gonad; i, intestine, p, pharynx; s, stomach. (B)Growing bud. TC14-3 (hatched coelomic space) is expressed evenly along the proximal-distal (P→D) axis of bud. (C)Bud 1 day after detached from the parent. TC14-3 disappears from the proximal coelomic space where cell proliferation begins. (D)2-day-developing bud. Gut and pharyngeal rudiments (gr, pr) appear. (E)1-week-old bud. It becomes a miniature of adult zooid. (F)Amino acid sequences of TC14-1, TC14-2 and TC14-3. N-terminal amino acids (1-20) are signal peptides. Asterisks show positions where amino acids are identical with one another. The elements of secondary structure are shown according to x-ray resolution of TC14-1 [19]. Boxed amino acids at positions 65, 69, 106, 113, and 114 are mainly dealt with in this study.

*Polyandrocarpa misakiensis *is a budding tunicate. Buds arise as outgrowths of the parent body wall (Figure 1A). Soon after detached from the parent, a bud begins morphogenesis restricted to the proximal area (Figure 1B-D), and in about a week, it becomes a miniature of adult zooid (Figure 1E). TC14-3 is a 14-kDa Ca^2+^-dependent, galactose-binding tunicate protein that is widely expressed in the coelomic space of bud (Figure 1B) [6]. Interestingly, TC14-3 disappears from the in vivo morphogenetic, proximal area of bud immediately before cell growth and differentiation begin (Figure 1C) [6]. TC14-3 is one of several different but closely related polypeptides isolated from *P. misakiensis *(Figure 1F) [6-8]. All TC14s belong to the C-type lectin family, which is characterized by a specific carbohydrate recognition domain (CRD) [9]. TC14-1 induces epithelial transformation of undifferentiated coelomic cells during budding in *P. misakiensis *[10]. TC14-2 can form a heterodimer together with TC14-3, although other biochemical features and biological functions are unknown [6]. TC14-3 exhibits cytostatic activities that regulate in vitro cell proliferation, cell adhesion, and cell differentiation of multipotent epithelial cells [6]. We wondered why only TC14-3 but not TC14-2 possesses these activities.

The Polycomb group (PcG) genes are involved in epigenetic control of genomic activity. PcGs in *Drosophila *were initially identified as homeotic gene repressors [11,12]. PcG proteins bind in vivo to many discrete sites on the chromosome [13]. In mammals, PcG homologs play a role in genome-wide gene silencing [14]. They are essential for cell fate maintenance in embryonic stem cells [15] and hematopoietic stem cells [16]. In keratinocytes, PcG proteins regulate cell growth, differentiation, and senescence [17]. Polycomb repressive complex 2 (PRC2), a biochemically discernible component of PcG, is involved in gene repression by histone modification [18]. PRC2 contains several core proteins: Histone H3 methyltransferase (Ezh2) catalyzes trimethylation of H3 at Lys27 (H3K27me3); Eed and Suz12 are Ezh2 activators [16]. We found recently that a *Polyandrocarpa *homolog of *Eed *(*PmEed*) was remarkably induced during budding, an expression pattern similar to that of the TC14s [6,10]. It seems, therefore, likely that *PmEed *is involved in the cytostatic activity of TC14-3.

In this study, we aimed to disclose why and how only TC14-3 exerts the unique cytostatic activity in *P. misakiensis*. First, we examined amino acid moieties responsible for the cell growth-inhibitory activity of TC14-3. Using chimeric and mutant proteins, we demonstrate that protein dimerization and Ca^2+ ^binding motifs are essential for the cytostatic activity of TC14-3. Second, downstream genes of TC14-3 were looked for, using wild-type and mutant proteins. We present evidence that *PmEed *is up-regulated in vivo and in vitro by wild-type TC14-3. In relation to *Eed *induction, we show immunocytochemically histone H3 trimethylation in *Polyandrocarpa *cell nuclei. Using RNA interference (RNAi), rescue experiments were done to demonstrate that *PmEed *mediates the cell growth-inhibitory activity of TC14-3. Taken together, budding tunicates provide us with a unique and interesting system in which a coelomic polypeptide can induce a PcG gene and epigenetic histone modification.

## Results

### Survey of functional domains for cytostatic activity of TC14-3

Figure 1F shows the alignment of TC14-1, TC14-2, and TC14-3 sequences. All 3 proteins are composed of 145 amino acids, of which 20 N-terminal amino acids are signal peptides. The remaining 125 amino acids constitute the mature protein. The CRD of TC14s consists of 2 α helices, 5 β strands, and 4 loops (Figure 1F) [19]. The second α helix (α2) spanning positions 56-69 contributes to protein dimerization, and loop 3, loop 4, and β4 strand form a calcium pocket for galactose and fucose recognition (Figure 1F) [7,19].

**Figure 2:**
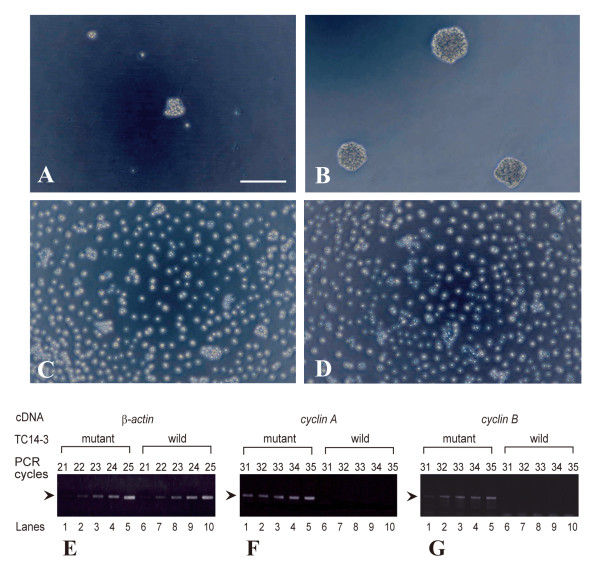
**Effects of wild-type and chimeric proteins on in vitro cell growth (A-D) and gene expression (E-G)**. Cells were plated and cultured for 2 days in the growth medium containing 30 μg/ml proteins. (A)Wild-type TC14-3. (B) TC14-2^21-60^/TC14-3^61-145^. (C)Wild-type TC14-2. (D)TC14-3^21-60^/TC14-2^61-145^. Bar, 50 μm. (E-G)Agarose gel staining of RT-PCR products (arrowheads). Lanes 1-5, TC14-3^E106G^. Lanes 6-10, wild-type TC14-3. (E)*Pmβ-actin*. (F)*PmCyclin A*. (G)*PmCyclin B*.

Two chimeric proteins containing complementary fragments from TC14-2 and TC14-3 were constructed (see Materials and methods). One of the chimeric proteins (TC14-2^21-60^/TC14-3^61-145^) consisted of N-terminal TC14-2 and C-terminal TC14-3. It reversibly blocked cell growth, similar to wild-type TC14-3 (Figure 2A, B). The other chimeric protein (TC14-3^21-60^/TC14-2^61-145^), like TC14-2, did not show such activity (Figure 2C, D), suggesting that the active site(s) for cell growth inhibition are located in the C-terminal region of TC14-3. In growth-arrested cells, the transcription of both *cyclin A *and *cyclin B *was suppressed (Figure 2E-G).

**Figure 3:**
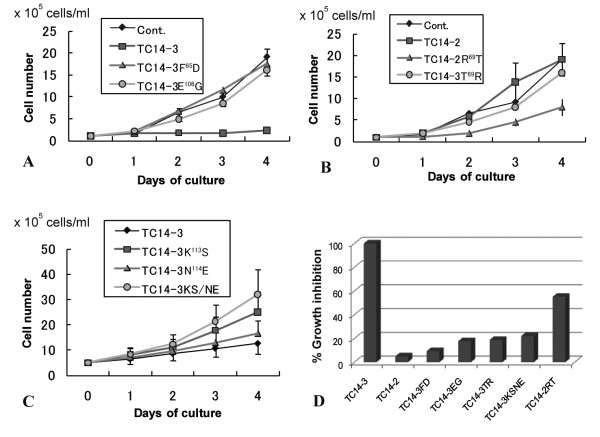
**Quantitative data of cell growth inhibition of wild-type and mutant TC14s**. Each histogram shows a mean ± standard deviation. (A)Effects of wild-type TC14-3, TC14-3^F65D^, and TC14-3^E106G ^on cell growth. (B)Effects of wild-type TC14-2, TC14-2^R69T^, and TC14-3^T69R ^on cell growth. (C)Effects of TC14-3^K113S^, TC14-3^N114E^, and TC14-3^K113S.N114E ^on cell growth. (D)Relative cell growth inhibition activities of aberrant TC14s as compared with 100% activity of wild-type TC14-3.

Next, we surveyed the polypeptide domains necessary for the cytostatic activity of TC14-3. Phe^65 ^in the α2 helix, Glu^106 ^in loop 3, and Asn^109 ^in loop 4 were changed to Asp, Gly, and Gly, respectively. TC14-3^F65D ^and TC14-3^E106G ^completely lost cytostatic activity (Figure 3A), and TC14-3^N109G ^exhibited lower activity (Table 1), suggesting that α2 helix and loop 3 are important for cytostatic activity. However, because both Phe^65 ^and Glu^106 ^are conserved in both TC14-2 and TC14-3 (Figure 1F), these amino acids are insufficient to explain the unique cytostatic activity of TC14-3.

**Table 1:** Summary of the cytostatic activities of mutant TC14-3s.

F^65^D	T^69^R	G^70^R	A^103^S	G^105^D	E^106^G	N^109^G	A^111^Q	G^112^S	K^113^S	N^114^E	K^113^SN^114^E	N^136^R	T^144^D	K^145^D
-	-	+++	+++	+++	-	++	+++	+++	++	++	+	+++	+++	+++

+++, Cytostatic activities remained strong.

++, Cytostatic activities faintly decreased.

+, Cytostatic activities were weakened to a large extent.

-, Cytostatic activities were almost lost.

### Amino acids involved in TC14-3-specific protein dimerization and cytostatic activity

**Figure 4:**
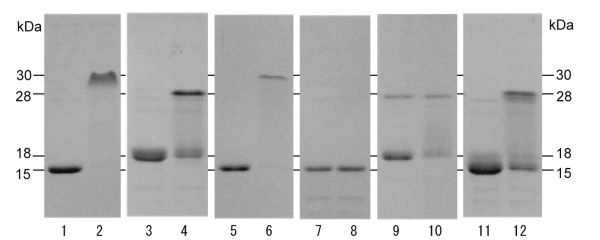
**Electrophoretic mobility of wild-type and mutant TC14s on SDS-PAGE**. Odd lanes and even lanes show heat-denatured samples and non-heated samples, respectively. Lanes 1,2, wild TC14-3. Lanes 3,4, wild TC14-2. Lanes 5,6, TC14-2^(21-60)^/TC14-3^(61-145)^. Lanes 7,8, TC14-3^F65D^. Lanes 9,10, TC14-3^T69R^. Lanes 11,12, TC14-2^R69T^.

TC14-3 exhibited a relative electrophoretic mobility of 15 kDa (Figure 4, lane 1) on SDS-PAGE following heat denaturation, while under non-heated conditions, more than 99% of the total protein exhibited a relative mobility of 30 kDa (Figure 4, lane 2; Table 2). In contrast, TC14-2 exhibited a single band of 18 kDa following heat denaturation (Figure 4, lane 3) and separated into 2 bands of 18 and 28 kDa under non-heated conditions (Figure 4, lane 4). The 28-kDa form of TC14-2 accounted for approximately 61% of the total amount of protein (Table 2). The chimeric protein, TC14-2^21-60^/TC14-3^61-145 ^exhibited an electrophoretic pattern similar to that of wild-type TC14-3 (Figure 4, lanes 5, 6). These results strongly suggest that wild-type TC14-3 may form more stable dimers than wild-type TC14-2.

**Table 2:** Relative amounts of monomeric and dimeric forms in wild-type TC14s and their mutant proteins.*

	Monomer (%)	Dimer (%)
TC14-3, wild	< 1.0	> 99.0

TC14-2, wild	38.9	61.1

TC14-3^F65D^	100	0

TC14-3^T69R^	33.3	64.2

TC14-2^R69T^**	15.7	69.3

*, SDS-PAGE was done under non-heated condition. After staining, each band was scanned with a gel scanner.

**, TC14-2^R69T ^had a few intermediate bands, so that the sum of monomer and dimer did not attain to 100%.

The mutant protein TC14-3^F65D ^failed to dimerize (Figure 4, lanes 7, 8, Table 2). At the extremity of the α2 helix (Figure 1F), Thr^69 ^of TC14-3 was exchanged with Arg^69 ^of TC14-2. Under heat denaturation, TC14-3^T69R ^exhibited a major band of approximately 18 kDa instead of 15 kDa (Figure 4, lane 9), and under the non-heated condition, it yielded 2 bands of 18 and 28 kDa (Figure 4, lane 10, Table 2), similar to wild-type TC14-2. On the other hand, heat-denatured TC14-2^R69T ^exhibited a major band of 15 kDa (Figure 4, lane 11), similar to wild-type TC14-3. In contrast, the non-heated sample of TC14-2^R69T ^yielded 2 bands of 15 and 28 kDa, intermediate between wild-type TC14-2 and TC14-3 (Figure 4, lane 12, Table 2).

TC14-3^T69R ^exhibited no cytostatic activity on cultured tunicate cells (Figure 3B,D). TC14-2^R69T^, on the other hand, acquired the cytostatic activity to some extent (Figure 3B,D). As a reference, the amino acid at position 70 was exchanged between TC14-2 and TC14-3. The cytostatic activity of the mutant proteins was unaffected (Table 1).

These results indicate that the amino acid at position 69 can modulate multiple characteristics of TC14s, such as electrophoretic mobility, stability of protein dimers, and cytostatic activity.

### Amino acids involved in TC14-3-specific Ca^2+ ^binding and cytostatic activity

**Figure 5:**
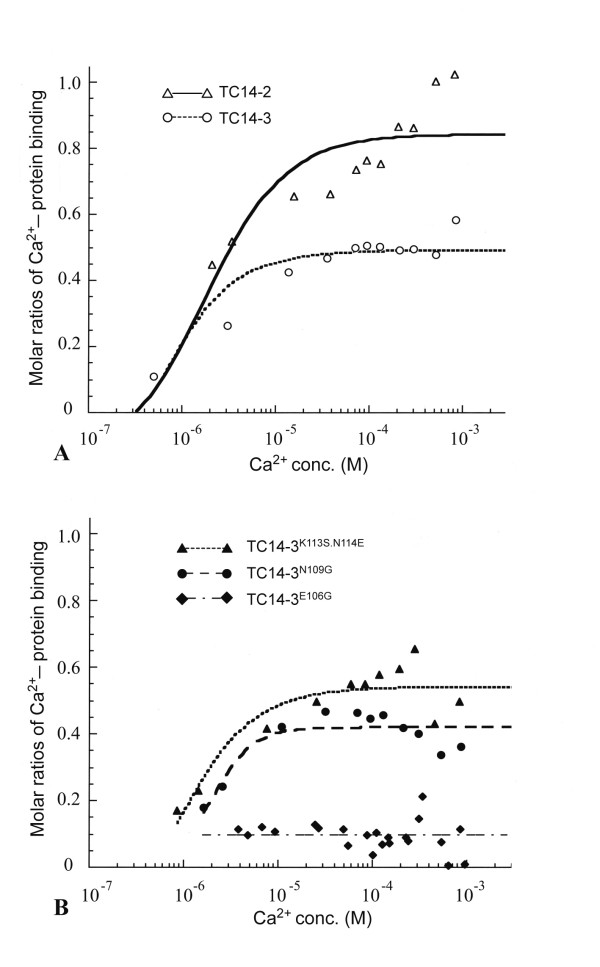
**Calcium-binding kinetics of wild-type and mutant TC14s**. (A)Wild type TC14-2 and TC14-3. Note that the Ca^2+^-binding affinity of TC14-3 is lower than that of TC14-2. (B)TC14-3^E106G^, TC14-3^N109G^, and TC14-3^K113S.N114E^. TC14-3^N109G ^showed lower Ca^2+^-binding affinity than wild-type TC14-3 and TC14-3^K113S.N114G ^showed the higher affinity to some extent.

Figure 5 shows the quantitative data of Ca^2+ ^binding in wild-type and mutant TC14s. Wild-type TC14-2 bound to calcium at a molar ratio of 1:0.85, while the calcium binding ratio of wild-type TC14-3 was unexpectedly low (1:0.5) (Figure 5A). TC14-3^E106G ^exhibited negligible Ca^2+^-binding activity (Figure 5B), and TC14-3^N109G ^exhibited reduced calcium-binding efficiency (molar ratio, 0.4) (Figure 5B). As mentioned, TC14-3^E106G ^lost the cytostatic activity near-completely, while TC14-3^N109G ^exhibited weak cell growth inhibition.

We next focused on the amino acids at positions 113 and 114 in loop 4 of TC14s (Figure 1F). Although single mutations (TC14-3^K113S ^or TC14-3^N114E^) did not improve calcium binding, the double mutation TC14-3^K113S.N114E ^bound to calcium at a molar ratio of approximately 0.6 (Figure 5B), a value intermediate between wild-type TC14-3 and wild-type TC14-2 (Figure 5A).

Both TC14-3^K113S ^and TC14-3^N114E ^retained their growth-inhibitory activities on cultured cells (Figure 3C). On the other hand, the inhibitory activity was greatly diminished in the double mutant protein TC14-3^K113S.N114E ^(Figure 3C,D). Mutations at C-terminal positions 136, 144, and 145 did not have any apparent influence on cell growth (Table 1).

### Only wild-type TC14-3 can induce PmEed

**Figure 6:**
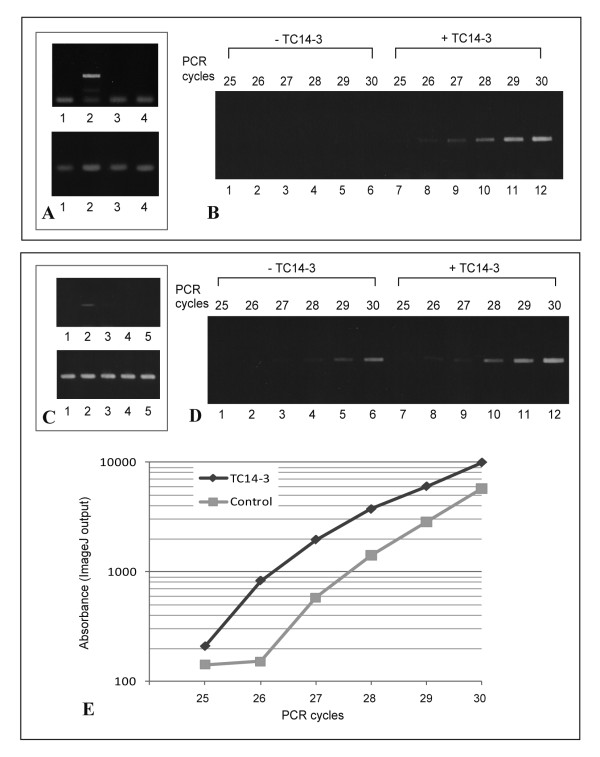
**Quantification of in vitro and in vivo *PmEed *induction by wild-type and mutant TC14-3s**. (A, B)Cultured cells. (A)PCR products of *PmEed *(upper) and *Pmβ-actin *(lower). Lane 1, control (PBS). Lane 2, wild-type TC14-3. Lane 3, TC14-3^T69R^. Lane 4, TC14-3^E106G^. (B)PCR products from 25^th ^to 30^th ^cycle. Lanes 1-6, control (PBS). Lanes 7-12, wild-type TC14-3. (C-E)Aged zooid pieces. (C)PCR products of *PmEed *(upper) and β-actin (lower). Lane 1, control (PBS). Lane 2, wild type TC14-3. Lane 3, TC14-3^K113S.N114E^. Lane 4, TC14-3^T69R^. Lane 5, TC14-3^E106G^. (D)PCR products from 25^th ^to 30^th ^cycle. Lanes 1-6, control (PBS). Lanes 7-12, wild-type TC14-3. (C)Increasing kinetics of PCR products quantified by ImageJ output.

We examined whether TC14-3 influenced the gene expression of *PmEed*. Cultured cells of *Polyandrocarpa *were treated for 2 days with PBS, wild-type TC14-3, TC14-3^T69R^, or TC14-3^E106G^. *PmEed *cDNA could be amplified by RT-PCR only when wild-type TC14-3 was applied to cells (Figure 6A). The amount of *PmEed *continued to increase during PCR cycles (Figure 6B).

**Figure 7:**
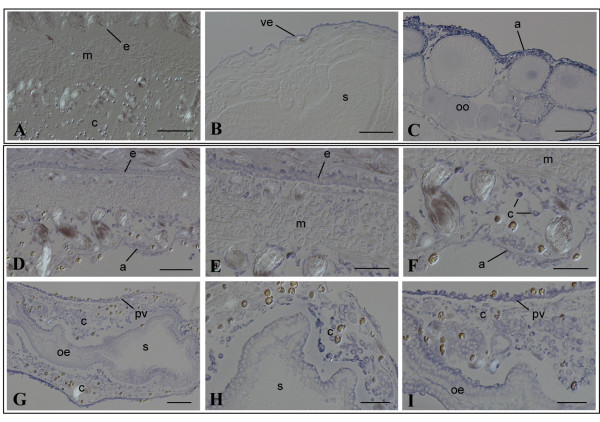
**In situ hybridization of *PmEed *in adult zooids treated with TC14-3**. (A-C)Control, PBS. (A)Body wall of zooid. Bar, 100 μm. (B)Digestive tract. Bar, 50 μm. (C)Gonad. Bar, 50 μm. (D-I)Experiment, wild-type TC14-3. (D)Body wall of zooid. Bar, 50 μm. (E)Epidermis and muscle cells. Bar, 25 μm. (F)Coelomic cells in the body wall. Bar, 25 μm. (G)Digestive tract. Bar, 50 μm. (H)Coelomic cells around the stomach. Bar, 25 μm. (I)Visceral epithelium. Bar, 25 μm. a, atrial epithelium; e, epidermis; c, coelomic cell; m, muscle cell; oe, oesophagus; oo, oocyte; s, stomach; pv, perivisceral epithelium.

In intact animals, *PmEed *was expressed abundantly from bud stages to juvenile zooid stages [see Additional file 1A, B, C, D], but diminished conspicuously at adult zooid stages except the gonad [see Additional file 1A, E, F] (More detailed results will be published elsewhere). In this study, adult zooids were cut into 3 pieces to facilitate TC14-3 infiltration, and treated with TC14-3 proteins for 2 days. Zooids of *P. misakiensis *possess a high potential for regeneration [20]. As expected, control zooid pieces treated with PBS could survive during the course of study. They did not exhibit any apparent signals for *PmEed *in most tissues and organs except the gonad (Figure 7A-C), similar to intact adult zooids, indicating that the surgery by itself did not affect *PmEed *expression. In contrast to the control, zooid pieces that had been treated with wild-type TC14-3 ubiquitously expressed *PmEed *(Figure 7D, G), the expression pattern similar to buds. The strongest signal was detected in coelomic cells in the hemocoel (Figure 7F, H, I). The atrial, gastric, and perivisceral epithelia also expressed *PmEed *(Figure 7F, I). The epidermis showed moderate expression of *PmEed*, but muscle cells did not (Figure 7E).

Results of RT-PCR showed that only wild-type TC14-3 could induce in vivo *PmEed *(Figure 6C). By semi-quantitative PCR, the *PmEed *products became visible at the 25^th ^cycle (Figure 6D), and increased exponentially thereafter (Figure 6D, E). In the control, on the other hand, *PmEed *products became first visible at the 27^th ^cycle (Figure 6D), and increased parallel to the experiment (Figure 6E). The result indicated that the amount of *PmEed *transcripts in wild-type TC14-3-treated animals was approximately 2-4-fold that of the control.

### TC14-3 also induces mitochondrial respiratory gene

**Figure 8:**
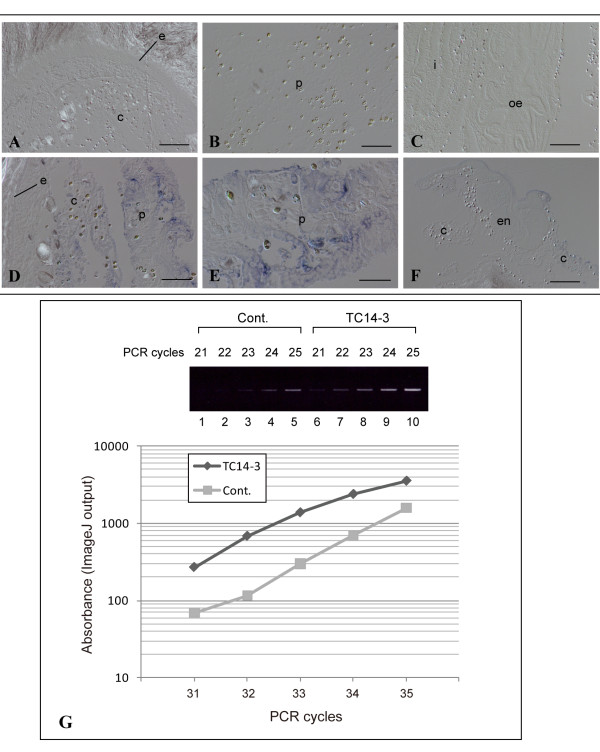
**In vivo *PmCOX1 *induction in adult zooids by TC14-3**. (A-F)In situ hybridization. (A-C)Control treated with PBS. (D-F)Experiment treated with wild-type TC14-3. (A)Body wall. Bar, 100 μm. (B)Pharynx. Bar, 50 μm. (C)Visceral organs. Bar, 100 μm. (D)Body wall and pharynx. Bar, 50 μm. (E)Pharynx. Bar, 25 μm. (F)Endostyle. Bar, 100 μm. c, coelomic cell; e, epidermis; en, endostyle; i, intestine; oe, oesophagus; p, pharynx. (G)Semi-quantitative PCR of *PmCOX1*. (Upper)Gel electrophoresis. β-actin was used as internal standards (see Figure 6). (Lower)Kinetics of increasing curve of PCR products.

Our recent study showed that in *P. misakiensis*, *PmEed *and mitochondrial respiratory genes were both inactivated during zooidal senescence and reactivated remarkably during budding (Kawamura et al., submitted). We examined, therefore, whether wild-type TC14-3 could induce not only *PmEed *but also *cytochrome c oxidase 1 *(*PmCOX1*) in aged zooids. Results of in situ hybridization showed that in the control, signals were hardly detectable in the body wall, pharynx, and visceral organs (Figure 8A-C). In contrast, when TC14-3 was applied to zooids, a portion of epithelial cells and coelomic cells in the pharynx expressed *PmCOX1 *strongly (Figure 8D, E). The endostyle, digestive tract, and surrounding coelomic cells did not emit signals (Figure 8F). The increasing curves of PCR products indicated that TC14-3-treated samples had larger amount of *PmCOX1 *transcripts than untreated controls, although the difference was not so high (Figure 8G).

### Trimethylation of histone H3 by TC14-3

**Figure 9:**
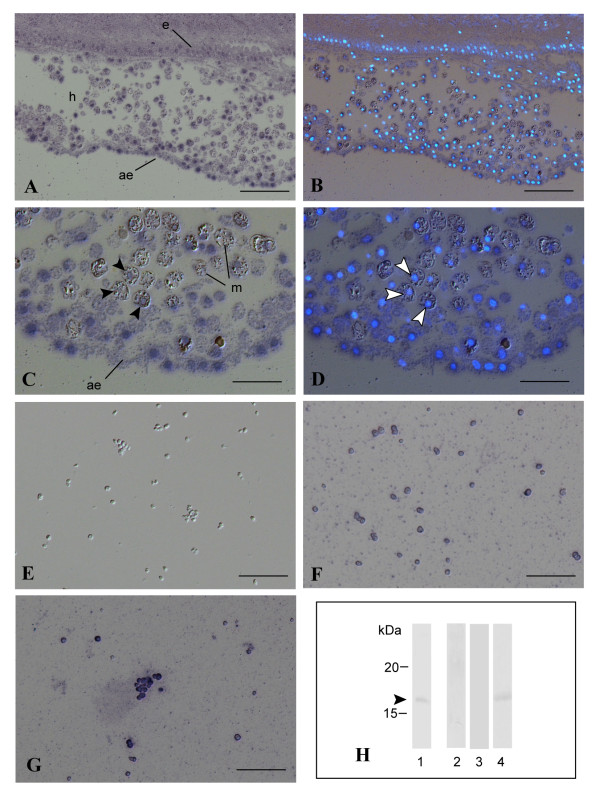
**In vivo and in vitro immunostaining of trimethylated histone H3 in *P. misakiensis***. (A-D)Growing buds stained with anti-H3K27me3 antibody. (B, D)DAPI staining after immunostaining. (A, B)Bars, 50 μm. (C, D)Bars, 25 μm. Black and white arrowheads show nuclei of morula cells. (E-G)Cultured cells stained with anti-H3K27me3 antibody. (E)Cells not treated with TC14-3. Bar, 50 μm. (F)Cells treated with TC14-3^E106G^. (G)Cells treated with wild-type TC14-3. Bar, 50 μm. (H)Western blotting of cell lysates. Lanes 1, anti-histone H3 antibody staining. Arrowhead shows histone H3. Lanes 2-4, anti-H3K27me3 antibody staining. Lane 2, cells not treated with TC14-3. Lane 3, cells treated with TC14-3^E106G^. Lane 4, cells treated with wild-type TC14-3. ae, atrial epithelium; e, epidermis; h, hemocoel; m, morula cell.

Anti-H3K27me3 antibody stained the in vivo nuclei of epithelial cells and coelomic cells in buds (Figure 9A, B). Nuclei of epidermal cells stained weakly (Figure 9A), whereas those of the atrial epithelium, multipotent epithelial cells in *P. misakiensis*, stained heavily (Figure 9A, C, D). In the hemocoel, many coelomic cells emitted strong signals (Figure 9A, C), but differentiated cells such as morula cells did not have apparent signals in the nucleus (Figure 9C black arrowheads, 9D white arrowheads).

Cultured cells untreated with TC14-3 were not stained with anti-H3K27me3 antibody (Figure 9E). Cells treated with mutant protein (TC14-3^E106G^) were stained weakly (Figure 9F), whereas wild-type TC14-3-treated cells were stained heavily with the antibody (Figure 9G). Western blotting of in vitro cultured cells showed that anti-histone H3 antibody stained a single band of approximately 17 kDa (Figure 9H, lane 1). Anti-H3K27me3 antibody, on the other hand, did not stain any bands when cells were not treated or treated with TC14-3^E106G ^(Figure 9H, lanes 2, 3), but stained a single band of 17 kDa when cultured cells were treated with wild-type TC14-3 (Figure 9H, lane 4). We could not find in vivo differences in histone trimethylation between TC14-3-treated and untreated samples (not shown).

The gene expression of *PmEzh2*, a *Polyandrocarpa *homolog of Histone H3K27 methyltransferase, was examined. Adult zooid fragments treated with wild-type TC14-3 showed the same strength of signals as those of untreated zooids [see Additional file 2 lanes 1, 2). Cultured cells in the growth medium without TC14-3 showed a weak signal of *PmEzh2 *PCR products at 30^th ^cycle [see Additional file 2 lane 3]. When cells were treated in vitro with wild-type or mutant TC14-3s, the signals were approximately the same as those of the control [see Additional file 2 lanes 4-7). These results indicate that wild-type TC14-3 can induce H3K27me3 without affecting *PmEzh2 *gene expression.

### Recovery from TC14-3-induced growth arrest by PmEed knockdown

**Figure 10:**
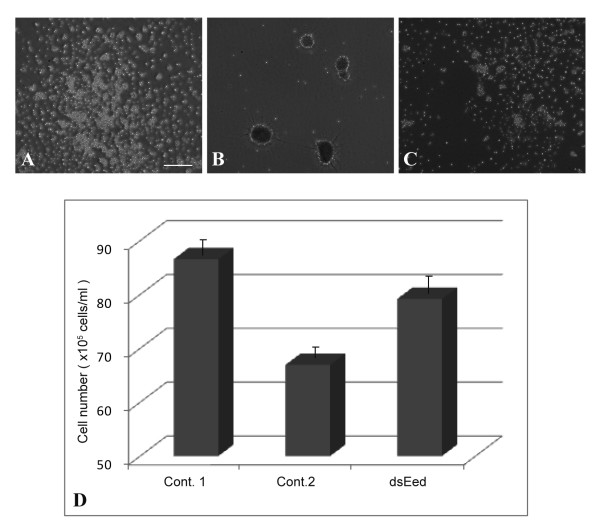
**Recovery of cell growth by RNAi of *PmEed *from TC14-3-induced growth arrest**. (A)In control 1 (positive control), after blunt electroporation cells were allowed to grow for 3 days in the absence of TC14-3. Cell spread normally. Bar, 100 μm. (B)In control 2 (negative control), cells were treated with TC14-3 after blunt electroporation. Cells formed many aggregates. (C)In dsEed (RNAi experiment), cells were treated with TC14-3 after electroporation of double-stranded RNA of *PmEed*. Cells spread again. (D)Quantification of cell number. In respective Cont. 1, Cont. 2, and dsEed, cell number was calculated from the standard curve of MTT assay. Each histogram shows a mean ± standard deviation.

We examined the effect of *PmEed *RNAi on cell growth arrest by wild-type TC14-3. Double-stranded RNA of *PmEed *(dsRNA*_PmEed_*) was introduced into cultured cells by electroporation. In the positive control, blunt electroporation was performed in the absence of dsRNA*_PmEed_*, and the cells were allowed to grow for 3 days without TC14-3. Cells spread on the culture dish (Figure 10A). In the negative control, cells were treated with TC14-3 after the blunt electroporation. Cells formed many aggregates (Figure 10B). In dsRNA*_PmEed _*experiments, cells spread again in the presence of TC14-3 (Figure 10C). The cell number was approximately twice as many as that of the negative control (Figure 10D). The recovery value accounted for 65% compared to the positive control.

## Discussion

### α2 helix and loop 3 are essential for the cytostatic activity of TC14-3

The results of the chimera experiments revealed that the amino acids at positions 61-145 in the C-terminal region of TC14-3 are responsible for cytostatic activity. The C-terminal region contains 1 α helix (α2), 4 β strands (β2-β5), and 4 loops (L1-L4) (see Figure 1F). In the α2 helix of TC14-1, hydrophobic amino acids (Ala^61 ^and Phe^65^) play a key role in protein dimerization [19]. Our study, using site-directed mutagenesis and SDS-PAGE of recombinant proteins, confirmed that in TC14-3, Phe^65 ^of α2 helix is essential for protein dimerization and also critical for cytostatic activity.

TC14s are Ca^2+^-binding proteins [7]. The ligands for calcium are the side-chain oxygen atoms of Glu^106 ^(loop 3), Asn^109 ^(loop 4), Asp^127 ^(β4 strand), and Asp^128 ^(β4 strand), as well as the main-chain carbonyl oxygen of Asp^128 ^(see Figure 1F) [19]. In TC14-3, Glu^106 ^of loop 3 played a key role in Ca^2+ ^binding, and the loss of Ca^2+ ^binding was associated with the loss of cytostatic activity. Glu^106 ^and Asn^109 ^of TC14s correspond to Glu^185 ^and Asn^187 ^of mannose-binding protein A (MBP-A), respectively. In MBP-A, double mutations, Glu^185^Gln and Asn^187^Asp, alter the sugar substrate specificity from mannose to galactose [21].

In E-selectin, the sequence Trp-Ala-Pro-Gly-Glu-Pro (76-81) regulates carbohydrate-binding specificity [22]. If Ala at position 77 is replaced with Ser, the sugar specificity of the mutant E-selectin changes from sialic acid to mannose. An exactly identical sequence exists in loop 3 of TC14-3 (see Figure 1F, positions 102-107). The corresponding sequence of TC14-2 was Trp-Ser-Pro-Asp-Glu-Pro. Both TC14-3^A103S ^and TC14-3^G105D ^retained strong cytostatic activity (see Table 1). It is, therefore, unlikely that the loop 3 is responsible for the difference between TC14-2 and TC14-3, although the loop 3 is essential for determining biological and biochemical features of TC14s.

Angiostatin and endostatin are specific, potent inhibitors of endothelial proliferation and angiogenesis [1,2]. Endostatin is a 20-kDa C-terminal fragment of collagen XVIII. TC14-3 is similar to endostatin in several respects. The X-ray structure of murine endostatin is similar to that of C-type lectin [23]. It lacks a characteristic Ca^2+^-binding site, but instead binds zinc at the N-terminus. This metal binding enables the dimerization of human endostatin [24]. Similar to TC14-3, protein dimerization is essential for endostatin to carry out the antitumor activity [3].

### Thr^69 ^modulates TC14-3 dimerization

TC14-3 differed from TC14-2 in protein dimer stability. As Phe^65 ^of α2 helix is conserved in both TC14-2 and TC14-3, we hypothesized that the differences in the biological and biochemical properties of TC14-2 and TC14-3 may consist in α2 helix neighboring Phe^65^.

The amino acids at position 69 of TC14-3 and TC14-2 are Thr and Arg, respectively. As Arg has a large side chain, it would interfere with the fitting and hydrophobic bonds at the α2 helix between juxtaposing proteins. As expected, TC14-3^T69R ^changed the electrophoretic mobility and the stability of protein dimers, and lost the cytostatic activity. In contrast, TC14-2^R69T ^could not form stable dimers comparable to that of wild-type TC14-3. This result suggests that additional as yet unidentified amino acids may contribute to the stability of protein dimers. However, it is undoubted that the amino acid at position 69 can modulate the biological and biochemical properties of TC14s.

### Lys^113 ^and Asn^114 ^modulate Ca^2+ ^binding of TC14-3

The cytostatic activities of TC14-3 depend on calcium-dependent galactose binding [6]. Therefore, we initially expected that the affinity of TC14-3 for calcium may be higher than that of TC14-2. However, contrary to our expectation, the Ca^2+^-binding affinity of TC14-3 was apparently lower than that of TC14-2.

Lys^113 ^and Asn^114 ^are specific for TC14-3. They are located at the boundary between loop 4 and the β3 strand. When both these amino acids were replaced with those of TC14-2, the resultant TC14-3^K113S.N114E ^exhibited an increase in Ca^2+^-binding affinity (> 0.6) and a decrease in cytostatic activity. As mentioned, TC14-3^N109G ^had low Ca^2+^-binding affinity (0.4), and exhibited reduced cytostatic activity. Taken together, TC14-3 appears to have the highest cytostatic activity when the binding ratio of protein to Ca^2+ ^is 1:0.5.

### PmEed mediates cytostatic activity of TC14-3

In *P. misakiensis*, the atrial epithelium is a transdifferentiation-competent, multipotent tissue [5,25]. It undergoes the terminal differentiation into the pharynx, gut, and brain when growing buds enter the developmental stage [25]. TC14-3 is induced remarkably during budding, and it disappears from the morphogenesis domain where transdifferentiation takes place [6]. This disappearance of TC14-3 may be caused by retinoic acid-inducible serine protease [26]. TC14-3 can block in vitro cell growth and differentiation in *Polyandrocarpa *cell lines that have been established from explants of the atrial epithelium [6,27]. Consequently, Matsumoto et al. [6] have argued that in *P. misakiensis*, TC14-3 serves as a negative regulator of terminal differentiation of multipotent cells.

In *P. misakiensis*, *PmEed *was developmentally regulated during budding cycle. The gene expression of *PmEed *was the highest at bud stages, gradually diminish during zooid growth, and was almost absent in somatic tissues and organs of adult zooids (Kawamura et al., submitted). This expression pattern was similar to that of TC14. In the present study, wild-type TC14-3 could induce *PmEed *in both cultured cells and adult zooid tissues, and interestingly, mutant proteins with abnormalities in protein dimerization or Ca^2+ ^binding failed to induce *PmEed*.

Semi-quantitative PCR analysis of zooid pieces revealed that in the presence of TC14-3, the amount of *PmEed *transcripts was 2-4-fold higher than that of the control. This value seemed smaller than that expected from the results of in situ hybridization. This may be due to strong signals from the gonads in the control as well as the experiment. In fact, many gonads are embedded in the ventral body wall (see Figure 1A), and they particularly expressed *PmEed *in adult tissues in a TC14-3-independent manner. Therefore, the net induction of PmEed may be much larger, if the background value in the gonad could be subtracted from the total signal.

In *P. misakiensis*, dsRNA*_PmEed _*rescued cultured cells from the growth-inhibitory effect of wild-type TC14-3. This result affords further evidence that *PmEed *is a downstream mediator of cytostatic TC14-3. In mammals, when Eed is deficient in ES cells, PcG target genes are de-repressed [14], leading to cell growth and differentiation. Therefore, PcG is thought to play roles in stem cell renewal and inhibition of cell differentiation in ES cells [15]. Our results are consistent with these findings and notion in mammals.

### Other genes regulated by TC14-3

A previous study has shown that in *P. misakiensis*, TC14-3 up-regulates α-integrin gene expression [6]. In this study, wild-type TC14-3 suppressed the gene expression of both *cyclin A *and *cyclin B*. In *Drosophila*, PcG directly down-regulates *cyclin A *[28].

In *P. misakiensis*, mitochondrial respiratory complex genes are regulated in accordance with *PmEed *during budding life cycle (Kawamura et al., submitted). When wild-type TC14-3 was applied to zooid pieces of *P. misakiensis*, *PmCOX1 *gene was up-regulated. This gene regulation may also be related to *PmEed*. However, it should be noted that, unlike *PmEed*, the expression of *PmCOX1 *was not ubiquitous, but restricted around the pharynx. It is, therefore, possible that mitochondrial respiratory complex genes may be up-regulated via a route other than *PmEed*.

### Epigenetic histone H3 trimethylation involved in cell growth and differentiation

Eed and Ezh2 are the components of PRC2 in PcG [18]. Eed acts as Ezh2 activator, and Ezh2 catalyzes H3K27me3 in the so-called histone tail [16]. Trimethylation of histone H3K27 recruits PRC1 to the chromatin. PRC1 possesses a discrete enzyme activity that modifies histone H2A, resulting in genome-wide, epigenetic gene repression [14]. *Polyandrocarpa *histone H3 showed 100% sequence similarity to mammalian histone H3.3 (not shown). Rabbit anti-histone H3K27me3 antibody indeed stained nuclei of the atrial epithelium and coelomic cells in intact buds of *P. misakiensis*. Our in vitro studies indicated that wild-type TC14-3 could induce H3K27me3 in *Polyandrocarpa *cultured cells. It is notable that TC14-3 up-regulated the *PmEed *gene expression, but not *PmEzh2*. Therefore, epigenetic trimethylation of histone H3K27 should be ascribable exclusively to enhanced *PmEed *gene expression.

In contrast with the atrial epithelium and coelomic cells, nuclei of epidermal cells and coelomic morula cells were stained very weakly with anti-H3K27me3 antibody. The epidermis is a specialized tissue to synthesize and secrete tunic components. Morula cells are differentiated cells engaged in self-defense mechanisms. In the light of multipotency of the atrial epithelium [5,25], it is probable that H3K27me3 is related to the block of terminal differentiation in budding tunicates. In ES cells, STAT3, Oct-3/4, and Sox2 induce Eed that influences H3K27me3 in the nucleus [29,30]. These transcription factors are essential for stem cell maintenance. Although the atrial epithelium in tunicates is quite different from ES cells in origin and developmental potential, the basic mechanism for keeping the multipotent cell state appears to be shared by tunicate cells and mammalian ES cells.

Trithorax group also modifies histone H3 by trimethylation of Lys4. However, the result of histone methylation is quite different from the case of PcG, making chromatin loose and activating differentiation genes [16]. In *P. misakiensis*, Lys4 trimethylation occurs in the process of transdifferentiation, which will be reported in the near future.

## Conclusions

As mentioned, TC14-3 is similar to endostatin in several aspects, but there are, of course, important differences between them. Endostatin binds α5β1 integrin and E-selectin on the endothelium [31] and inhibits the activity of metalloproteinases [32]. TC14-3, on the other hand, exerts cell growth inhibition at least in part by inducing in vivo and in vitro *PmEed*. A major function of induced *PmEed *is to facilitate H3K27me3. This system of budding tunicates consisting of a humoral factor, PcG, and histone trimethylation can regulate cell growth and differentiation of multipotent cells. Consequently, the homeostatic maintenance of transdifferentiation-competent cells would support budding and regenerative activities in *P. misakiensis*. Further studies of how humoral growth inhibitors such as endostatin and TC14-3 work in dimerization- and cation-dependent manners will afford insight into therapeutic control of malignant and/or multipotent cells and tissues.

## Methods

### Animals

Asexual individuals of *P. misakiensis *were reared in culture boxes placed in the Uranouchi Inlet near the Usa Marine Biological Institute, Kochi University.

### Cell culture and bioassay

*Polyandrocarpa *cells were cultured as described previously [27]. Cells were harvested in cell dissociation medium (0.2% trypsin and 2 mM EDTA in DMEM). They were resuspended in the growth medium at a density of 1 × 10^5 ^cells/ml, and 100 μl of this solution was plated in each well of a 96-well multiplate. Recombinant TC14s were added to the cell suspension at a final concentration of 30 μg/ml. As a control, sterile PBS (10 μl) was added to each well. Cells were counted with a hemocytometer [6] or the 3-[4,5-dimethylthiazol-2-yl]-2,5-diphenyl tetrazolium bromide (MTT) method [33]. For in vivo bioassay, adult animals were cut transversely into 3 pieces and incubated for 2 days in sterile seawater in the presence or absence of 30 μg/ml of wild-type TC14-3.

### cDNAs and site-directed mutagenesis

TC14-2 [DDBJ, AB049564], TC14-3 [DDBJ, AB049565], PmEed [DDBJ, AB617630], and PmEzh2 [DDBJ, AB671227] were used. Inverse PCR for mutagenesis was done using LA Taq DNA polymerase (Takara Bio Inc., Otsu, Japan): 1 cycle at 94°C for 1 min; 30 cycles at 94°C for 1 min, 55°C for 1 min, and 72°C for 4 min; and 1 cycle at 72°C for 4 min. PCR products were treated with T4 polymerase for 5 min to produce blunt ends. After the phosphorylation of the 5' end by polynucleotide kinase (Takara Bio Inc.), linear DNAs were made circular by DNA ligase (Takara Bio Inc.). Mutation was confirmed by DNA sequencing.

### Chimeric TC14s

In both TC14-2 and TC14-3, a unique *Hind*III restriction site was created at amino acid positions 60-62 by site-directed mutagenesis [see Additional file 3]. After the digestion with restriction enzymes, 3' fragments of TC14-2 and TC14-3 were exchanged with each other, and were ligated to 5' fragments. The chimeric cDNAs were mutated again to restore the original KAI sequence [see Additional file 3].

### DNA sequencing

For cycle sequencing, the Thermo Sequenase Dye Terminator cycle sequencing premix kit (Amersham Pharmacia Biotech., Piscataway, NJ, USA) was used. The products were analyzed using a DNA sequencer (373A; ABI, Foster City, CA, USA).

### Preparation of recombinant proteins

Glutathione *S*-transferase (GST)-TC14 fusion proteins were prepared as described previously [6]. Briefly, cDNAs were subcloned into pGEX vector (Amersham Pharmacia Biotech), and expressed in the bacterial strain BL21. Proteins were induced with 0.1 mM isopropyl-β-D-thiogalactopyranoside (IPTG), solubilized by sonication in a protein lysis buffer (6 M urea, 2 mM EDTA, and 0.2 mM dithiothreitol [DTT] in 0.1 M Tris-HCl [pH 8.0]), and dialyzed against phosphate-buffered saline (PBS). TC14s were eluted with 1 μg/ml thrombin from GST fusion proteins bound to glutathione beads (Amersham Pharmacia Biotech).

### Electrophoresis

Sodium dodecyl sulfate-polyacrylamide gel electrophoresis (SDS-PAGE) was performed with the method of Laemmli [34]. Proteins were treated with SDS sample buffer with or without heat denaturation. After electrophoresis, the gels were stained with Coomassie Brilliant Blue G250.

### Antibodies

Mouse anti-histone H3 antibody (05-499) and rabbit anti-histone H3K27me3 antibody (07-449) were purchased from Upstate, Millipore Corp. (Temecula, CA, USA). Secondary antibodies labeled with horseradish peroxidase were purchased from Vector Laboratory (Burlingame, CA, USA). Immunohistochemistry and western blotting were done as described previously [6], except that the primary antibody was preincubated with keyhole limpet hemocyanin (0.3 mg/ml) for 5 min to prevent nonspecific staining. Specimens or nitrocellulose membrane were colored by Trueblue (KPL, MD, USA).

### Gel scanning

After acrylamide gel staining, the proteins were scanned with Kodak EDAS 290 (Eastman Kodak Ltd., Rochester, NY, USA). The staining intensity of each band was quantified using Image Analysis software (ver. 3.5) (Eastman Kodak Ltd.). PCR products were separated by agarose gel electrophoresis and stained with ethidium bromide. They were scanned and quantified using ImageJ free software developed by the National Institutes of Health.

### Ca^2+ ^binding experiments

Protein-Ca^2+ ^binding was measured by the flow dialysis method, using ^45^CaCl_2 _(Amersham Pharmacia Biotech, CA, USA) in 0.1 M NaCl, 20 mM MOPS (pH 7.0) at 25°C. The protein concentration was adjusted to 25-75 μM. The loss of radioactive ligands during experiments and the nonspecific Ca^2+ ^binding to the apparatus were corrected. The resulting Ca^2+ ^binding data were analyzed by the Adair-Klots equation for a single binding site.

### Semiquantitative PCR

Poly(A)^+ ^RNA was extracted and purified from cultured cells and adult zooids by the biotinyl magnet method, according to the manufacturer's protocol (Roche, Mannheim, Germany). Single-stranded DNA complementary to poly(A)^+ ^RNA was synthesized for 1 h at 42°C using StrataScript reverse transcriptase (Agilent Technologies, Santa Clara, CA, USA). The DNA pool was stored as templates for PCR. PCR was performed in 2 steps: 1 cycle for sense strand synthesis (30 s at 94°C, 2 min at 52°C, and 2 min at 72°C); 24-35 cycles of denaturation for 30 s at 94°C, annealing for 60 s at 52°C, and extension for 90 s at 72°C. As an internal standard, β-actin cDNA was amplified by PCR.

### In situ hybridization

The protocol for in situ hybridization has been described previously [35]. In brief, specimens were fixed in 4% paraformaldehyde in PBS at 4°C for 10-16 h. The fixed specimens were rinsed in PBS containing 0.1% Tween 20 (PBST), digested with proteinase K, and postfixed in 4% paraformaldehyde and 1% glutaraldehyde in PBST. Specimens were hybridized with digoxigenin-labeled antisense RNA probe for 12-14 h at 58°C. After thorough washing, samples were incubated in blocking solution (1% skim milk in Tris-buffered salt solution containing 0.1% Tween 20) for 6 h in an ice bath, and then treated overnight on ice with anti-digoxigenin monoclonal antibody labeled with alkaline phosphatase (Roche, Mannheim, Germany). The samples were stained with the color development solution, dehydrated, and embedded in Technovit 8100 resin (Heraeus Kulzer, Wehrheim, Germany).

### Double-stranded RNA

*PmEed *cDNA (approximately 1 kb) devoid of poly(A) tail was inserted into pGEM-T (Promega Co.). Using the T7 RNA polymerase transcription system, sense and antisense RNA strands were synthesized. Both RNA solutions were mixed and heat-denatured for 10 min at 95°C. Then, the temperature was gradually lowered to anneal the double-stranded RNA (dsRNA). Immediately before use, the dsRNA was dissolved in RNase-free seawater at the final concentration of 0.5 μg/ml.

### Electroporation

Cells were harvested using cell dissociation solution. After washing, the cells were resuspended in HEPES-buffered salt solution (pH 7.2) at a density of 1 × 10^5 ^cells/ml. After a 10-min incubation of cells with dsRNA, electroporation was performed in a 2-mm cuvette with a pulse of 200 V and 100 μF using GENE pulser Xcell (BioRad, USA). After 10 min, cells were transferred to the growth medium.

## List of abbreviations

COX1: cytochrome c oxidase 1; CRD: carbohydrate recognition domain; Eed: embryonic ectoderm development (Esc homolog); Ezh2: enhancer of zeste homolog 2; H3K27me3: trimethylation of H3 at Lys27; PcG: polycomb group; PCR: polymerase chain reaction; PRC: polycomb repressive complex; RNAi: RNA interference; SDS-PAGE: sodium dodecyl sulfate-polyacrylamide gel electrophoresis; TC14: tunicate calcium-dependent, galactose-binding protein.

## Authors' contributions

KK prepared and assayed chimeric proteins, carried out gene cloning and expression analysis of *PmEed*, *PmEZH2*, and *PmCOX1*; KT purified mutant proteins and analyzed protein dimerization; DM designed site-directed mutagenesis and analyzed Ca^2+ ^binding domains; KI carried out RNAi and rescue experiments; AN carried out Ca^2+^-binding kinetics; TS participated in the discussion concerning the structural biology of mutant proteins. All authors read and approved the final manuscript.

## Supplementary Material

Additional file 1 **Expression of *PmEed *during asexual life span in *P. misakiensis***. (A)RT-PCR of *Pmβ-actin *(lanes 1-3) and *PmEed *(lanes 4-6). Lanes 1,4, Growing bud. Lanes 2,5, Juvenile (2- or 3-week-old) zooid. Lanes 3,6, Adult zooid. (B-F)In situ hybridization of *PmEed*. (B)Growing bud, distal tip. Bar, 50 μm. (C, D)Juvenile zooid. (C)Pharynx. Bar, 50 μm. (D)Ventral body wall. Bar, 25 μm. (E, F)Adult zooid. (E)Intestine and surrounding perivisceral epithelium. Bar, 50 μm. (F)Gonad. Bar, 50 μm. c, coelomic cell; e, epidermis; i, intestine; o, oocyte; p, pharynx. Click here for file

Additional file 2 **Semi-quantitative PCR of *PmEzh2 *in adult zooids (lanes 1,2) and cultured tunicate cells (lanes 3-7) treated with TC14-3s**. Lanes 1,3, control (PBS). Lane 2,4, wild type TC14-3. Lane 5, TC14-3^T69R^. Lane 6, TC14-3^E106G^. Lane 7, TC14-3^K113S.N114E^. Click here for file

Additional file 3 **Experimental procedure for chimeric protein production**. Both cDNA of TC14-2 and TC14-3 were mutated at the position Ile^61 ^to make an unique site for *Hind *III (top). They were cut with *Hind *III to exchange the C-terminal fragments with each other (middle). After ligation, chimeric cDNAs were mutated again to change Phe61 to Ile61 before transferred to expression vectors (bottom). Click here for file
