# Supplementary figures and images for: Tunicate cytostatic factor TC14-3 induces a polycomb group gene and histone modification through Ca2+ binding and protein dimerization
Source: BMC Cell Biol. 2012 Feb 2;13:3. doi: 10.1186/1471-2121-13-3 (PMC3293724; doi:10.1186/1471-2121-13-3)

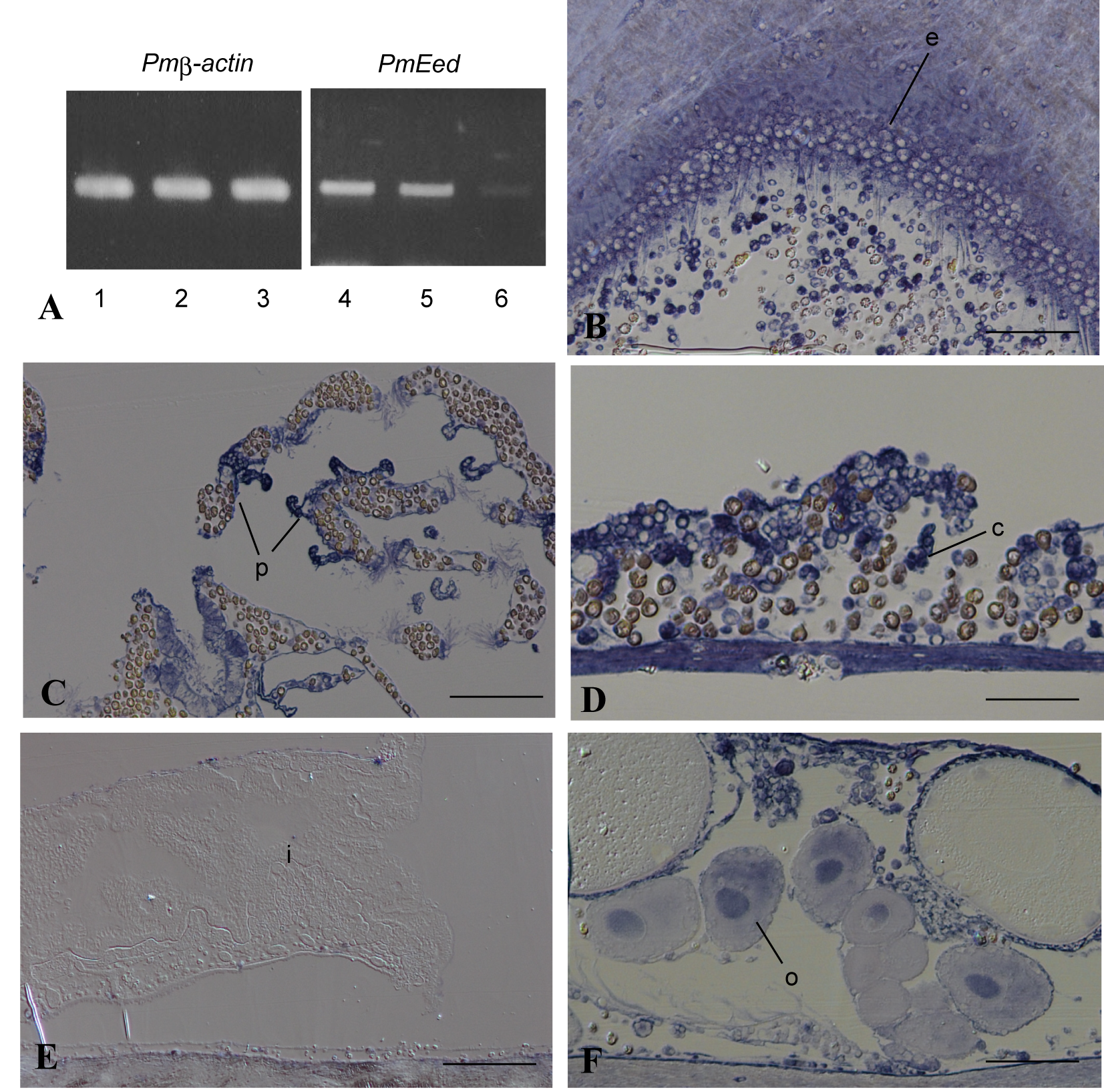

Supplement: Additional file 1 — Expression of PmEed during asexual life span in P. misakiensis. (A)RT-PCR of Pmβ-actin (lanes 1-3) and PmEed (lanes 4-6). Lanes 1,4, Growing bud. Lanes 2,5, Juvenile (2- or 3-week-old) zooid. Lanes 3,6, Adult zooid. (B-F)In situ hybridization of PmEed. (B)Growing bud, distal tip. Bar, 50 μm. (C, D)Juvenile zooid. (C)Pharynx. Bar, 50 μm. (D)Ventral body wall. Bar, 25 μm. (E, F)Adult zooid. (E)Intestine and surrounding perivisceral epithelium. Bar, 50 μm. (F)Gonad. Bar, 50 μm. c, coelomic cell; e, epidermis; i, intestine; o, oocyte; p, pharynx. [file 1471-2121-13-3-S1.TIFF]

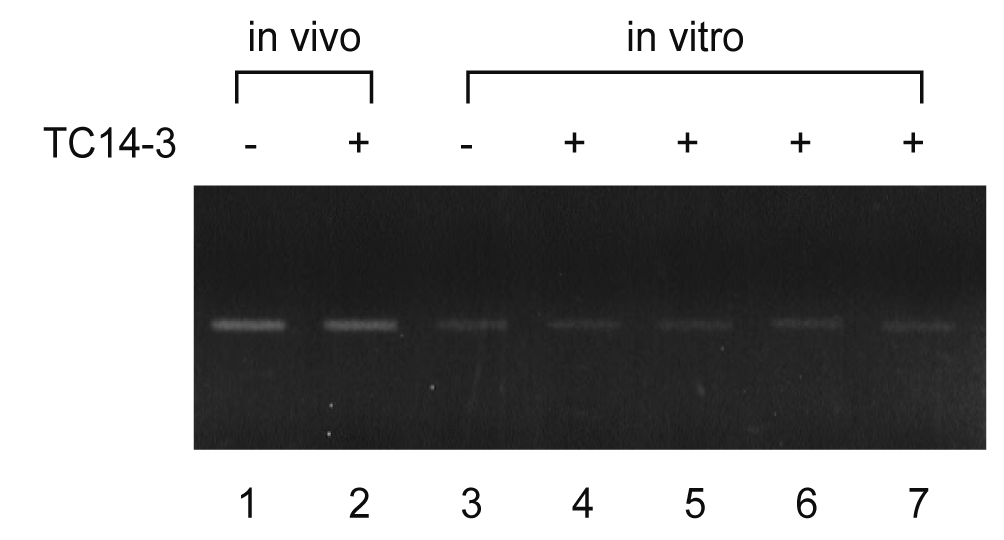

Supplement: Additional file 2 — Semi-quantitative PCR of PmEzh2 in adult zooids (lanes 1,2) and cultured tunicate cells (lanes 3-7) treated with TC14-3s. Lanes 1,3, control (PBS). Lane 2,4, wild type TC14-3. Lane 5, TC14-3T69R. Lane 6, TC14-3E106G. Lane 7, TC14-3K113S.N114E. [file 1471-2121-13-3-S2.TIFF]

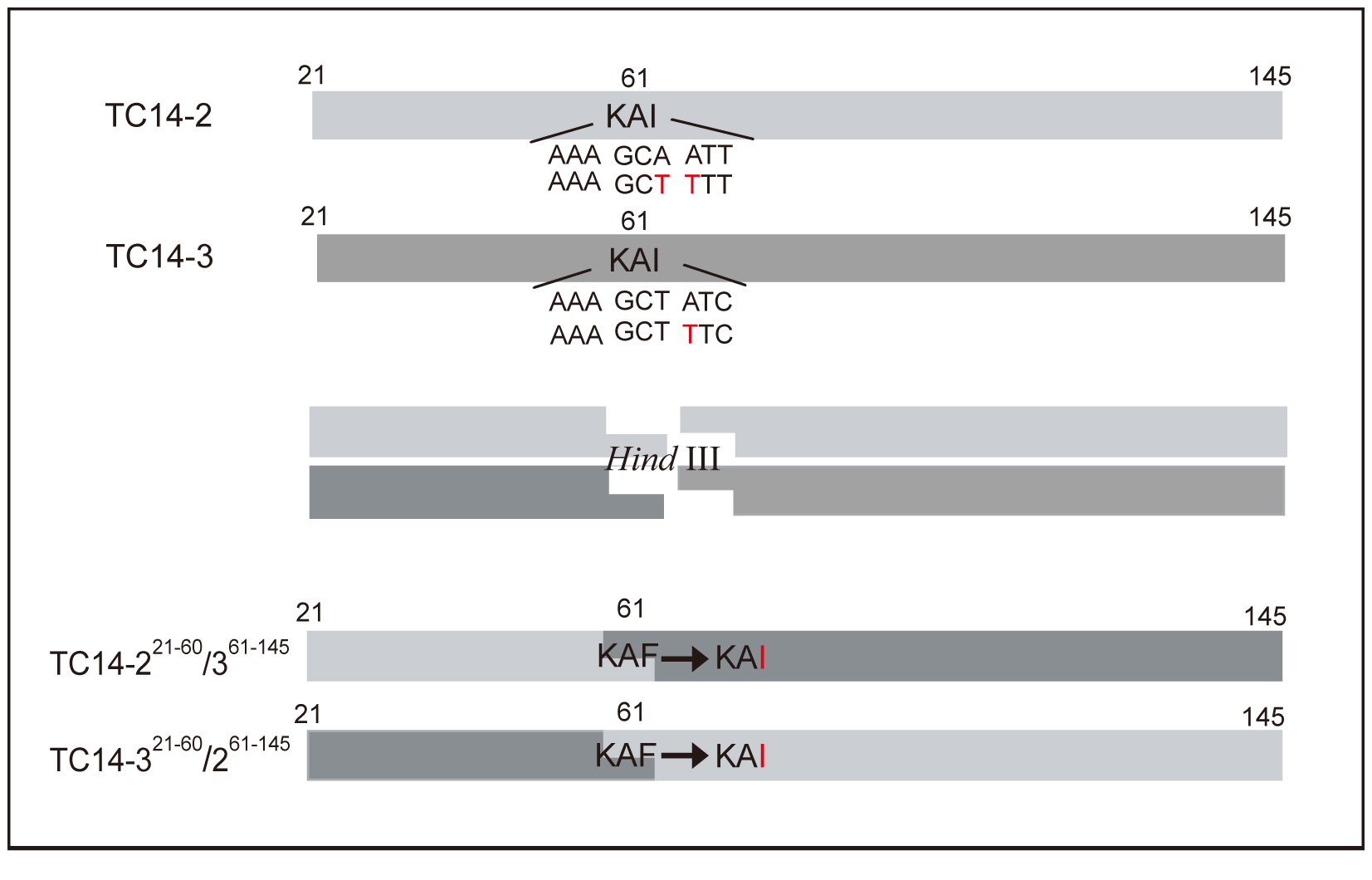

Supplement: Additional file 3 — Experimental procedure for chimeric protein production. Both cDNA of TC14-2 and TC14-3 were mutated at the position Ile61 to make an unique site for Hind III (top). They were cut with Hind III to exchange the C-terminal fragments with each other (middle). After ligation, chimeric cDNAs were mutated again to change Phe61 to Ile61 before transferred to expression vectors (bottom). [file 1471-2121-13-3-S3.JPEG]
